# Medial Gastrocnemius Muscle Architecture Is Altered After Exhaustive Stretch-Shortening Cycle Exercise

**DOI:** 10.3389/fphys.2019.01511

**Published:** 2019-12-20

**Authors:** Adam Kositsky, Dawson J. Kidgell, Janne Avela

**Affiliations:** ^1^Biology of Physical Activity, Neuromuscular Research Center, Faculty of Sport and Health Sciences, University of Jyväskylä, Jyväskylä, Finland; ^2^Department of Physiotherapy, School of Primary and Allied Health Care, Faculty of Medicine, Nursing and Health Sciences, Monash University, Melbourne, VIC, Australia

**Keywords:** fatigue, ultrasound, fascicle length, pennation, stretch reflex, afferent, force

## Abstract

Muscle architecture is an important component of muscle function, and recent studies have shown changes in muscle architecture with fatigue. The stretch-shortening cycle is a natural way to study human locomotion, but little is known about how muscle architecture is affected by this type of exercise. This study investigated potential changes in medial gastrocnemius (MG) muscle architecture after exhaustive stretch-shortening cycle exercise. Male athletes (*n* = 10) performed maximal voluntary contractions (MVC) and maximal drop jump (DJ) tests before and after an exercise task consisting of 100 maximal DJs followed by successive rebound jumping to 70% of the initial maximal height. The exercise task ceased upon failure to jump to 50% of maximal height or volitional fatigue. Muscle architecture of MG was measured using ultrasonography at rest and during MVC, and performance variables were calculated via a force plate and motion analysis. After SSC exercise, MVC (−13.1%; *p* = 0.005; *d_z_* = 1.30), rebound jump height (−14.8%, *p* = 0.004; *d_z_* = 1.32), and ankle joint stiffness (−26.3%; *p* = 0.008; *d_z_* = 1.30) decreased. Ankle joint range of motion (+20.2%; *p* = 0.011; *d_z_* = 1.09) and MG muscle-tendon unit length (+12.0%; *p* = 0.037; *d_z_* = 0.91) during the braking phase of DJ, the immediate drop-off in impact force (termed peak force reduction) (Δ27.3%; *p* = 0.033; *d_z_* = 0.86), and lactate (+9.5 mmol/L; *p* < 0.001; *d_z_* = 3.58) increased. Fascicle length increased at rest (+4.9%; *p* = 0.013; *d_z_* = 1.16) and during MVC (+6.8%; *p* = 0.048; *d_z_* = 0.85). Pennation angle decreased at rest (−6.5%; *p* = 0.034, *d_z_* = 0.93) and during MVC (−9.8%; *p* = 0.012; *d_z_* = 1.35). No changes in muscle thickness were found at rest (−2.6%; *p* = 0.066; *d_z_* = 0.77) or during MVC (−1.6%; *p* = 0.204; *d_z_* = 0.49). The greater MG muscle-tendon stretch during the DJ braking phase after exercise indicates that muscle damage likely occurred. The lower peak force reduction and ankle joint stiffness, indicative of decreased active stiffness, suggests activation was likely reduced, causing fascicles to shorten less during MVC.

## Introduction

The architecture of a skeletal muscle describes the structural component that influences its function (Lieber and Fridén, 2000). The force-length relationship and the direction of muscle fiber force transmitted to the line of muscle action are imperative components of muscle mechanics. Muscle architecture can be visualized *in vivo* utilizing B-mode ultrasonography, and this methodology has been used over the past few decades to assess key architecture parameters, i.e., muscle fascicle length (FL) and pennation angle (PA), in various conditions (Franchi et al., 2018).

As fatigue is defined as a reduction in maximal force production resulting from exercise (Taylor and Gandevia, 2008), muscle architecture measures during maximal voluntary contraction (MVC) may reveal important information about muscle function immediately after exercise. Indeed, acute changes in muscle architecture have previously been reported in the human triceps surae after various exercise tasks. In particular, increased FL of medial gastrocnemius (MG) during an active contraction has been observed following repeated maximal isometric (Mitsukawa et al., 2009; Thomas et al., 2015) and eccentric contractions (Guilhem et al., 2016). In contrast, fascicles were shorter after repeated isometric contractions at submaximal levels (Mademli and Arampatzis, 2005). Nonetheless, prolonged exercise appears to have an effect on muscle architecture.

However, the immediate effects on muscle architecture after stretch-shortening cycle (SSC) exercise are less clear. The SSC is a crucial and inherent function that exists in human locomotion, whereby preactivated muscle-tendon units (MTU) lengthen eccentrically prior to concentric shortening (Komi, 2000). Because the SSC sequence occurs in many common activities, such as running and jumping, repetitive SSC exercise better represents naturally occurring fatigue compared to repetitive isolated isometric, concentric, or eccentric tasks (Komi, 2000; Nicol et al., 2006). Although fatiguing SSC exercise has been reported to alter MG (Lidstone et al., 2016; Kubo and Ikebukuro, 2019) and soleus (Ishikawa et al., 2006) muscle architecture, neither study took measures during MVC immediately post-exercise. Further, while there was increased length of soleus fascicles at rest following SSC exercise (Ishikawa et al., 2006), to our knowledge there are no data available regarding fatigue-related changes in resting MG muscle architecture after SSC exercise. Therefore, the purpose of the present study was to investigate the effect of exhaustive SSC exercise on MG muscle architecture at rest and during MVC. Based on the results of previous experiments, it was hypothesized that the exercise task would cause increases in FL and decreases in PA in both conditions. Performance parameters were also measured during MVC and SSC tasks.

## Materials and Methods

### Subjects

Ten male soccer players [mean age = 18.9 ± 0.9 years (all >18 years); mean height = 182.3 ± 6.2 cm; mean body mass = 75.9 ± 6.6 kg] were recruited. Subjects were requested to not perform any strenuous activity beginning 48 h prior to testing. The University of Jyväskylä Ethical Committee, in accordance with the Declaration of Helsinki, approved the study. Each subject was informed of the benefits and risks of the study and provided written consent prior to the commencement of the investigation.

### Procedures

At least one week prior to the fatigue protocol, subjects visited the lab for a familiarization session. Subjects were introduced to the sledge apparatus (Aura and Komi, 1986) inclined 24.9° from horizontal and the optimal dropping height for drop jumps (DJs) was determined (Avela et al., 2006). After being instructed to rebound jump as high and as fast as they could, subjects were dropped from 10 cm intervals and the rebound jump height was recorded. Dropping heights were increased until the rebound height no longer improved, and the dropping height with the corresponding highest rebound height was determined to be the optimal dropping height. Subjects were also shown how to perform and practiced MVCs of the plantarflexors on the sledge apparatus.

The second session consisted of the fatigue protocol on the sledge apparatus with MVC and maximal DJ measurements prior to, and ~5 min after, exercise. These post-exercise tests were not completely immediate as lactate and resting muscle architecture measurements were conducted first. Subjects performed a standardized, moderate-intensity warm-up on a cycle ergometer for 10 min (Regueme et al., 2005). Subjects then performed 100 successive maximal bilateral DJs (one DJ every 5 s; controlled with an audible metronome) from the optimal dropping height (Kuitunen et al., 2002; Ishikawa et al., 2006; Dousset et al., 2007), split into 5 sets of 20 with 2 min rest given between sets (Miyama and Nosaka, 2004). Immediately after the 100th maximal DJ, the subjects began continuous submaximal rebounding to a height corresponding to 70% of the initial maximal rebound height (Kuitunen et al., 2002; Ishikawa et al., 2006; Dousset et al., 2007). This was necessary because blood lactate does not accumulate after 100 sledge DJs in athletes (Avela et al., 2006). Rebound jumping continued until either 50% of maximal rebound height could not be attained or volitional fatigue. Subjects were given audible feedback regarding rebound jump height. To maximize fatigue of the triceps surae, subjects were not allowed to contact the force platform with their heels and were instructed to limit their knee angle to a maximum of 90° during ground contact (Regueme et al., 2005). During all jumps, the seat was inclined to 120° and subjects were advised to allow their knees to passively flex freely during the rebound airborne phase to reduce fatigue of the hip and knee extensors (Dousset et al., 2007).

### Performance Measures

A rigid, built-in force plate located at the bottom of the sledge apparatus recorded ground reaction force perpendicular to the movement plane of the sledge (Aura and Komi, 1986). Data were sampled at 1,000 Hz using Spike2 software (version 6.17, Cambridge Electronics Design, Cambridge, UK). MVCs were performed with subjects seated in the sledge apparatus. To maximize activation of the plantarflexors, heels were kept off the force plate by placing the distal, plantar surface of the feet over a footplate. During MVCs, adjustable metal bars held the sledge in a locked position with the knees in full extension and the ankles neutral. Data were taken as an average of 500 ms around the peak value and subtracted by baseline force (average of 500 ms prior to contraction) to exclude each subject’s body weight. Subjects were given 2 min rest between trials and the average of two trials was used.

As a performance test, subjects performed maximal bilateral DJs from the optimal dropping height with the same instructions and joint angle restrictions as in the familiarization session and fatigue protocol. Arms were folded across their chest to limit upward propulsion by the upper body. Jump height was recorded by marking the highest position of the sledge’s potentiometer and subtracting the subject’s relative standing height on the sledge. Upon landing, there is a large impact spike preceding a drop-off in force (Komi, 2000), termed peak force reduction, which was calculated as the difference between the peak impact force and the immediate lowest force level (Avela et al., 1999b). All jumps were recorded from the subject’s right side using a high-speed camera (Sony NXCAM, HXR-NX5E, Japan) at 200 frames per second. Reflective markers were placed over the greater trochanter, lateral femoral condyle, lateral malleolus, heel, and base of the fifth metatarsal of the right lower limb. Markers were digitized and processed (Vicon Motus 10.0, Vicon Motion Systems, Oxford, UK) with a 20 Hz low-pass Butterworth filter applied to the raw coordinates. Estimates of MG MTU length were derived from kinematic data using the equation of Hawkins and Hull (1990) and normalized to lower leg length (lateral femoral condyle to lateral malleolus). Videos were synchronized using an LED light that brightened when force increased above baseline levels. Subjects were given 2 min rest between trials and the average of 2–3 trials were used.

The ankle joint moment was estimated using the equation of Kawakami et al. (2002). Briefly, the formula accounts for the perpendicular ground reaction force, the estimated length between the center of the ankle joint (center of the lateral malleolus) and the ball of the foot, and the ankle joint angle at the lowest point (transition from braking to propulsion phase). Ankle joint stiffness was calculated as the change in ankle joint moment divided by the change in ankle joint angle during the braking (eccentric) phase (Kuitunen et al., 2002; Kubo and Ikebukuro, 2019).

Fingertips were cleansed with sanitizing alcohol before blood samples were drawn, and lactate was analyzed instantly using an automated device (Lactate Scout, SensLab GmbH, Germany). This analysis was conducted prior to warm-up and immediately upon completion of the fatiguing task.

### Muscle Architecture

**Figure 1:**
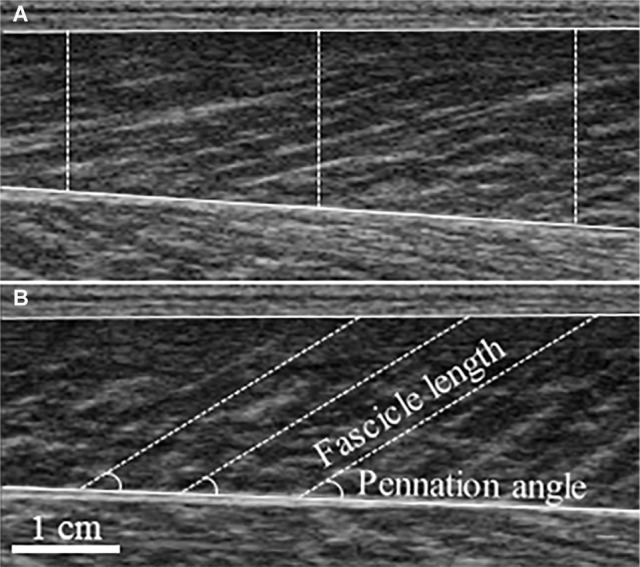
Typical ultrasound images of the medial gastrocnemius taken at rest **(A)** and during maximal voluntary contraction **(B)**. Measurement of muscle thickness is depicted by dashed vertical lines superimposed on the resting image, with fascicle length and pennation angle superimposed on the maximal voluntary contraction image.

Real-time B-mode ultrasonography (SSD-α10, Aloka, Japan) recorded longitudinal images of MG (Figure 1). The imaging location was determined by scanning the muscle belly for the thickest region where fascicles could be clearly seen from superficial to deep aponeurosis. A 7.5 MHz transducer (scanning length: 60 mm) was longitudinally attached over the skin of the right leg using a custom-made foam holder and elastic bandages. Acoustic coupling gel was applied between the transducer surface and skin. Resting images were taken with the subjects resting prone on a plinth with the ankle neutral. Images were obtained at 94 Hz and for MVCs synchronized with an electronic pulse on the force recordings that signaled video onset. FL was determined as the length of an ultrasonic echo, parallel to the muscle fascicles, between superficial and deep aponeuroses (Fukunaga et al., 1997) and extrapolated where necessary (Thomas et al., 2015; Guilhem et al., 2016). PA was measured as the angle between the fascicle and deep aponeurosis. The vertical distance between superficial and deep aponeuroses, defined as muscle thickness (MT), was averaged over three locations (left, center, right) of each image. Because MG images taken at rest have previously been established to be reliable (Kwah et al., 2013), FL and PA were taken as the average of three separate fascicles from a single image. For PA and MT during MVC, a still image was taken from the absolute peak force from the trials used in MVC force data. Data from 2–3 fascicles from two images and videos were averaged. Using a reliable, automated fascicle tracking algorithm (Matlab version R2016b, The MathWorks Inc., Massachusetts, USA) (Cronin et al., 2011), active FL was averaged over the synchronized and corresponding 500 ms window from trials that were used for peak MVC force analysis. All data were manually checked and corrected if necessary. For single images, open-source computer software (ImageJ 1.51h, National Institutes of Health, USA) was used.

### Statistical Analysis

Statistical analyses were performed on absolute values using IBM SPSS Statistics 25 (SPSS Inc., Chicago, USA). A Shapiro-Wilk test was performed to assess normal distribution. Two-tailed paired-sampled *t*-tests were used to assess differences from baseline measurements. As PA data during MVC were not normally distributed, a Wilcoxon signed-rank test was conducted instead. Statistical significance was set at *p* < 0.05 and confidence intervals at 95%. Cohen’s *d_z_* effect sizes (within-subjects design) were calculated from absolute values (Lakens, 2013) and interpreted as small ≥0.20, medium ≥0.50, and large ≥0.80.

One subject was excluded from all data as his post MVC value was considerably increased (~17%). It is possible that the optimal dropping height was determined incorrectly, inducing some facilitatory responses (Nicol et al., 2006). A second subject was excluded from ultrasound data due to errors in the recording. For a third subject, the braking phase could not be synchronized with the force signal during post-exercise DJ testing; thus, ankle joint stiffness could not be calculated, and he was excluded from this parameter. Finally, the reflective marker over the greater trochanter could not be accurately tracked for a fourth subject during the pre-exercise DJ trials. Therefore, knee joint range of motion and MG MTU length could not be determined, and he was excluded from these parameters. This resulted in *n* = 8 for the ultrasound, ankle joint stiffness, knee joint range of motion, and MG MTU length measures, and *n* = 9 for all other variables.

## Results

Performance parameters are summarized in Table 1 and demonstrate that fatigue was clearly induced. After exercise, significant reductions were shown in MVC (−13.1%; *p* = 0.005; *d_z_* = 1.30), rebound jump height (−14.8%, *p* = 0.004; *d_z_* = 1.32), and ankle joint stiffness (−26.3%; *p* = 0.008; *d_z_* = 1.30). Peak force reduction (Δ27.3%; *p* = 0.033; *d_z_* = 0.86) and lactate (mean difference: +9.5 mmol/L; *p* < 0.001; *d_z_* = 3.58) were significantly increased. During the maximal DJ test after exercise, only the change in ankle joint range of motion during the braking phase was significantly different (+20.2%; *p* = 0.011; *d_z_* = 1.09). The change in ankle joint range of motion during the propulsion phase (+2.3%; *p* = 0.459; *d_z_* = 0.26), and the change in knee joint range of motion during the braking (+2.1%; *p* = 0.647; *d_z_* = 0.17) and propulsion (−3.9%; *p* = 0.097; *d_z_* = 0.68) phases were not significantly different. This resulted in a significant difference in MG MTU length change during the braking (+12.0%; *p* = 0.037; *d_z_* = 0.91), but not the propulsion (−0.6%; *p* = 0.826; *d_z_* = 0.08), phase after exercise.

**Table 1:** Performance parameters before and after exhaustive exercise.

	Pre	Post
Maximal voluntary contraction (N)	1375.5(328.9)	1195.7(322.8)^**^
Rebound jump height (cm)	79.5(11.1)	67.7(11.6)^**^
Peak force reduction (N)	−746.3(201.8)	−949.7(253.0)^*^
Ankle joint stiffness (Nm/°)	7.36(1.98)	5.42(1.52)^**^
Ankle joint ROM braking phase (°)	38.6(8.3)	46.4(8.9)^*^
Ankle joint ROM propulsion phase (°)	55.8(8.3)	57.1(7.9)
Knee joint ROM braking phase (°)	62.7(13.3)	64.0(12.4)
Knee joint ROM propulsion phase (°)	88.1(6.1)	84.6(9.2)
MG MTU length change braking phase (mm)	52.6(8.9)	58.9(9.2)^*^
MG MTU length change propulsion phase (mm)	−74.1(11.3)	−73.6(10.7)
Lactate (mmol/L)	1.8(0.3)	11.3(2.7)^***^

* *(p < 0.05)*;

** *(p ≤ 0.01)*;

*** *(p < 0.001) from pre-exercise measures*.

*MG, Medial gastrocnemius; MTU, Muscle-tendon unit; ROM, Range of motion*.

*Joint angle changes are in absolute values. For MTU length changes, positive and negative values refer to lengthening and shortening, respectively*.

*Means with standard deviations in parentheses*.

Significant changes in muscle architecture were apparent post-exercise. FL was significantly longer at rest (+4.9%; *p* = 0.013; *d_z_* = 1.16) and during MVC (+6.8%; *p* = 0.048; *d_z_* = 0.85) (Table 2). Significant decreases were found for PA in both resting (−6.5%; *p* = 0.034, *d_z_* = 0.93) and MVC (−9.8%; *p* = 0.012; *d_z_* = 1.35) conditions (Table 2). There was no change in MT at rest (−2.6%; *p* = 0.066; *d_z_* = 0.77) or during MVC (−1.6%; *p* = 0.204; *d_z_* = 0.49).

**Table 2:** Muscle architecture measures at rest and during maximal voluntary contraction (MVC) before and after exhaustive exercise.

	Rest		MVC	
	Pre	Post	Pre	Post
Fascicle length (mm)	51.7 ± 7.0	54.3 ± 7.5^*^	26.2 ± 2.1	28.0 ± 4.1^*^
Pennation angle (°)	14.3 ± 2.2	13.4 ± 2.8^*^	31.9 ± 2.7	28.8 ± 4.8^*^
Muscle thickness (mm)	14.7 ± 1.4	14.4 ± 1.3	14.9 ± 1.0	14.7 ± 1.2

* *(p < 0.05) from pre-exercise measures*.

*Means with standard deviations in parentheses*.

## Discussion

The purpose of the study was to investigate changes in MG muscle architecture after exhaustive SSC exercise on a sledge apparatus. The results show increased FL and decreased PA with large effect sizes. Further, the changes in FL (rest: +4.9%; MVC: +6.8%) and PA (rest: −6.5%; MVC: −9.8%) were greater than the intra-session minimal detectable changes in gastrocnemius FL (4.8%) and PA (1.8%), as reported by Cè et al. (2015). There were no significant differences in MT either at rest or MVC. Additionally, the exercise task induced significant decrements to MVC and DJ performance.

### Muscle Architecture Changes at Rest

The increased range of motion at the ankle, but not knee, joint during DJ after exercise resulted in a greater stretch of the MG MTU during the braking phase. Because Achilles tendon mechanical properties are typically unchanged after prolonged SSC exercise (Peltonen et al., 2010, 2012; Lichtwark et al., 2013; Kubo and Ikebukuro, 2019), this likely resulted in changes at the muscle fascicle level. In non-fatigued conditions, MG fascicles generally shorten before operating quasi-isometrically or minimally stretching during DJs from the optimal dropping height, but undergo a larger stretch during DJs from heights greater than optimal (Ishikawa et al., 2005; Sousa et al., 2007). Indeed, Kubo and Ikebukuro (2019) have recently reported a large reduction in the amount of MG fascicle shortening during the braking phase of ankle-joint only DJs after repeated hopping. In particular, at group level the fascicles showed an initial stretch immediately after ground contact (Figure 1B in Kubo and Ikebukuro, 2019), demonstrating potential for fascicles to be exposed to greater strain as the exercise protocol proceeded. Greater fascicle strain as the exercise task progressed would have led to muscle damage (Lieber and Fridén, 1993; Peñailillo et al., 2015) that has previously been reported after similar fatigue protocols (Miyama and Nosaka, 2004; Ishikawa et al., 2006; Dousset et al., 2007). Damage to the non-contractile elements has been attributed to the increase in resting soleus fascicle length immediately after a similar SSC protocol (Ishikawa et al., 2006). While we did not find increases in MT, a measure that is associated with muscle damage, increases in MT do not always occur immediately after muscle damaging SSC exercise (Ishikawa et al., 2006; Dousset et al., 2007). Therefore, intramuscular pressure likely did not immediately change, and thus the resting pennation angle became smaller owing to space constraints within the muscle. Further, although muscle damage has recently been associated with increased passive stiffness (Lacourpaille et al., 2017), due to the constraints imposed by our experimental set-up, we were only able to test a surrogate of active stiffness (explained in the section “Limitations”). Nonetheless, longer MG fascicles at rest as a result of muscle damage is supported by a previous report of increased resting FL of soleus, in combination with greater passive stiffness, immediately post-exhaustive, muscle damaging SSC exercise (Ishikawa et al., 2006). Future studies should investigate the relationship of resting FL changes with changes in passive stiffness measures (e.g., shear modulus, passive torque) and muscle damage.

### Muscle Architecture Changes During Maximal Voluntary Contraction

Whereas passive stiffness may have increased, active stiffness likely decreased, as evidenced by reduced joint stiffness (Kuitunen et al., 2002; Kubo and Ikebukuro, 2019), greater peak force reduction (Avela et al., 1999b), and MVC output (Morel et al., 2019), all of which were found in the current study. Myoelectric activity, which is a major factor in active stiffness (Aura and Komi, 1986), has previously been shown to be decreased during MVC and maximal DJs after exhaustive SSC exercise (Avela et al., 1999b; Kuitunen et al., 2002; Ishikawa et al., 2006; Dousset et al., 2007). Reduced activation leads to fascicles being longer during contraction by way of less fascicle shortening (Avela et al., 2004; Bennett et al., 2014), which was not directly measured in the current study (explained in the section “Limitations”). Further, as fascicles shorten during contraction, the insertion points on the superficial and deep aponeuroses are pulled closer together, increasing pennation angle (Narici et al., 1996). Decreased activation would result in fascicle insertions being pulled together less, in turn causing less pennation change and thus a smaller overall pennation angle (Narici et al., 1996; Manal et al., 2008), which we did observe.

Although this could explain the reduction in fascicle shortening reported by Thomas et al. (2015), we, however, do not agree with those authors’ suggestion that longer fascicles would be a countermeasure from the central nervous system in an attempt to maintain optimal force in a fatigued state (Thomas et al., 2015). Increased FL of MG after fatigue has previously been reported to have an inverse relationship with torque, whereby subjects with greater FL had lower plantarflexion torque during MVC (Mitsukawa et al., 2009). This negative correlation may be because the longer fascicles are on the descending limb of the force-length relationship; however, this would clearly be detrimental for force production and not a strategy used by the central nervous system to preserve force in a fatigued state. Rather, Mitsukawa et al. (2009) also reported a decrease in electromyography amplitude concomitantly with increased MG FL as fatigue progressed. This supports a reduced muscle activation with fatigue resulting in less fascicle shortening during MVC.

As the Ia afferent system contributes to the active stiffness component and the impairment of the short-latency reflex (SLR) after exhaustive SSC is well established (Nicol et al., 2006), we cannot exclude the potential role that the intrafusal muscle fibers (i.e., Ia afferents), which are known to be activated and contribute to voluntary isometric contractions (Vallbo et al., 1979; Macefield et al., 1993), may have on this reduced fascicle shortening. As fatigue progresses, the discharge of muscle spindles decreases, reducing the output of alpha motoneurones during isometric contractions (Bongiovanni and Hagbarth, 1990; Macefield et al., 1991). A reduction in spindle support to alpha motoneurones would reduce activation level and force production and thus fascicles would shorten less during MVC. Although Day et al. (2017) found tibialis anterior FL and muscle spindle firing rate to follow the same pattern during passive, slow ankle rotations, and spindle length is largest when muscle length is also greatest (Figure 3 in Hoffer et al., 1989), spindle feedback rather than spindle lengthening appears to be affected by fatigue (Bongiovanni and Hagbarth, 1990; Macefield et al., 1991; Avela et al., 1999b). In that case, muscle spindles may be increased in length yet less responsive, dissociating the link between muscle FL and spindle discharge. Future studies should confirm this and additionally investigate the relationship between the amount of depressed SLR sensitivity and FL changes, as measuring FL could be a simpler way of determining reflex impairment in a fatigued state.

Contrary to our findings, Mademli and Arampatzis (2005) reported decreased FL of MG after a submaximal MVC fatiguing task. However, the exercise task was a sustained isometric contraction at 40% of MVC (Mademli and Arampatzis, 2005), an intensity likely too low to induce muscle damage. Further, Mademli and Arampatzis (2005) also reported increased MG myoelectric activity as fatigue progressed, even after tendon creep occurred, suggesting that there was at least some increase in muscle activation at the end of the exercise that could contribute to greater fascicle shortening. Thus, changes in muscle architecture are likely different depending on if the fatiguing task was of maximal or submaximal intensity.

### Implications for Performance

Ankle joint angle during the eccentric, braking phase is the denominator in the equation for ankle joint stiffness (Kuitunen et al., 2002; Kubo and Ikebukuro, 2019). Therefore, a greater change in ankle joint range of motion, as found in the current study, is also shown by a decrease in ankle joint stiffness, which needs to be high for efficient SSC performance (Hoffrén et al., 2007; Yoon et al., 2007). Indeed, the reduction in ankle joint stiffness during DJs after fatiguing hops was recently shown to be strongly related to the increase in ankle joint angle (*r* = −0.892), but not peak ankle moment (*r* = 0.318), which was unchanged (Kubo and Ikebukuro, 2019). In the current study, rebound jump height and ankle joint stiffness were reduced after exercise, suggesting that there is an optimal range of ankle joint motion during the eccentric, braking phase of SSC tasks that allows for maximizing SSC performance. Furthermore, the presence of high amounts of lactate post-exercise clearly suggests metabolic fatigue occurred. Because chemical factors are not always the main cause of the impaired SLR (Avela et al., 1999a), we did not specifically measure peripheral fatigue; however, metabolic fatigue stimulates the group III and IV afferents (Garland and Kaufman, 1995; Taylor and Gandevia, 2008). These small-diameter afferents can increase presynaptic inhibition of the Ia fibers (Bigland-Ritchie et al., 1986; Nicol et al., 2006), decreasing the contribution of the SLR to active stiffness during DJs and further hampering intrafusal fiber contribution to MVC. Therefore, maintaining active stiffness components appears to be essential for both SSC and MVC performance.

## Limitations

Not using electromyography to directly measure myoelectric activity and the SLR was a limitation in the present study; however, the decrements in performance parameters are comparable with those of previous reports using near-identical fatiguing protocols and impaired muscle activation and SLR function as a result of exhaustive jumps is clearly established (Nicol et al., 2006). Furthermore, although tendon slack length has been suggested to affect muscle architecture measurements (Aeles et al., 2017), the pre-post within-subjects study design likely negated these effects as Achilles tendon mechanical properties generally do not change after SSC exercise (Peltonen et al., 2010, 2012; Lichtwark et al., 2013; Kubo and Ikebukuro, 2019). Although a recent study demonstrated decreased Achilles tendon stiffness after a prolonged run at submaximal speed (Fletcher and MacIntosh, 2018), this would reduce the tension on the muscle fascicles, potentially shortening rather than lengthening them. Therefore, although tendon properties were unmeasured, they would be unlikely to explain the changes in muscle architecture in the current study. Additionally, FL at rest (prone) and during MVC (on the sledge) was measured in two different positions, which was necessary as the study set-up did not allow for the use of an isokinetic dynamometer. As subjects were “resting” on the sledge against body weight, we chose not to measure FL immediately prior to MVC. As such, comparing the changes from rest to MVC in two different positions could have led to errors when determining the amount of fascicle shortening and muscle bulging and thus were not reported.

## Conclusion

In summary, this study investigated the effects of exhaustive SSC exercise on MG muscle architecture, demonstrating increased FL, decreased PA, and no changes in MT both at rest and during MVC. The changes at rest are likely due to exercise-induced muscle damage and the changes during MVC are seemingly a result of reduced activation. Additionally, there appears to be an optimal range of ankle joint range of motion that allows for maximizing DJ rebound height. These results have important implications for better describing how human muscle adapts to fatigue and how SSC performance is optimized.

## Data Availability Statement

The raw data supporting the conclusions of this article will be made available by the authors upon reasonable request.

## Ethics Statement

This study involving human participants was reviewed and approved by the University of Jyväskylä Ethical Committee. The subjects provided their written informed consent to participate in this study.

## Author Contributions

AK and JA conceived and designed the research. AK conducted experiments, analyzed and interpreted data, and wrote the initial manuscript draft. DK and JA assisted in data interpretation and manuscript revisal. All authors read and approved the final manuscript.

### Conflict of Interest

The authors declare that the research was conducted in the absence of any commercial or financial relationships that could be construed as a potential conflict of interest.
